# Early Oral Colonization of Candidal Species in Young Children With Cleft Lip and Palate

**DOI:** 10.7759/cureus.104216

**Published:** 2026-02-25

**Authors:** Podile Sravani, K Ajay Benarji, Sreeja Jami, Vootla Naveen Reddy, K Vishnu Priya, Sindhura Gundapaneni, Avinash Illa

**Affiliations:** 1 Department of Oral Pathology, Drs. Sudha & Nageswara Rao Siddhartha Institute of Dental Sciences, Chinoutpalli, IND; 2 Department of Oral and Maxillofacial Surgery, University of Washington School of Dentistry, Seattle, USA; 3 Department of Dentistry, Government Medical College, Kothagudem, IND; 4 Department of Oral and Maxillofacial Surgery, Sri Siddhartha Dental College & Hospital, Tumkur, IND; 5 Department of Public Health Dentistry, University of California, Davis, USA

**Keywords:** caries severity index (csi), cleft lip, cleft lip and palate, early childhood dental caries, oral candida colonization

## Abstract

Background: Cleft lip and palate may promote early oral *Candida* colonization and caries. This study compared *Candida *carriage and oral health indices in 0-3-year-old children with trans-foramen cleft lip and palate versus age- and sex-matched controls.

Methods: In this observational study, 40 cleft and 40 control children were enrolled. Multi-site oral swabs were cultured with speciation (germ tube test for *Candida albicans* and CHROMagar for non-albicans *Candida*); fungal burden was reported as colony-forming units per mL. The def index and caries severity index (CSI) were analyzed using the chi-square test, independent t-tests, and Mann-Whitney U test.

Results: *Candida* carriage was higher in cleft children than controls (35 (87.5%) vs 8 (20.0%)), and growth categories differed (p<0.001). *C albicans* and non-albicans *Candida* were detected in 28 (70.0%) and 7 (17.5%) of cleft children versus 6 (15.0%) and 2 (5.0%) of controls. No clinical oral candidiasis was observed. Growth category was not associated with age band or sex within either cohort (all p>0.05). def was higher in the cleft group (5.62±0.35 vs 4.83±0.45; mean difference 0.79; p<0.001). The CSI was higher (2.79±0.33 vs 1.92±0.28; mean difference 0.87; p<0.001). Ordinal growth ranks favored the cleft cohort (mean rank 53.83 vs 27.18; p<0.001).

Conclusions: Early childhood cleft lip and palate was associated with substantially higher asymptomatic *Candida* colonization and worse caries-related indices, supporting cleft-adapted hygiene counseling and preventive dental surveillance rather than empiric antifungal therapy in asymptomatic children.

## Introduction

Children born with cleft lip and palate experience a distinctly altered oral ecosystem from the first months of life because anatomical discontinuity, feeding difficulties, plaque-retentive niches, and repeated clinical interventions can collectively impair routine hygiene and reshape early microbial maturation. Consistent epidemiologic signals indicate that children with clefts carry a higher burden of plaque-related disease, with elevated caries risk and variable enrichment of potentially pathogenic taxa, even though results differ by phenotype, sampling site, and laboratory approach [1-4]. Within this broader dysbiosis framework, the early-life mycobiome has become increasingly relevant because fungi can establish rapidly, interact with bacterial pioneers, and potentially influence subsequent cariogenic trajectories [5].

*Candida *species are among the most prevalent oral fungi in children, yet carriage is not uniform across infancy and early childhood and appears sensitive to exposures that modify the oral environment. In healthy pediatric cohorts, oral yeast carriage has been reported around one-quarter overall, with patterns varying by age and with signals that exclusive breastfeeding may be protective in the youngest subgroup [6]. Longitudinal mother-infant work further demonstrates that early acquisition of *Candida albicans* frequently reflects maternal strain concordance, with maternal plaque burden emerging as a modifiable correlate of vertical transmission [7]. Importantly, early *Candida *colonization has also been linked with accelerated emergence of Streptococcus mutans during infancy, supporting a biologically plausible bridge between fungal carriage and later cariogenic biofilm development [8].

In cleft populations, multiple studies report substantially higher oral *Candida* colonization than in non-cleft peers, with suggestions that cleft severity, bilateral phenotypes, prior surgeries, and appliance-related stagnation areas may amplify carriage or shift species profiles [9-12]. Perioperative antisepsis can transiently suppress colonization, yet follow-up data highlight dynamic patterns of elimination, persistence, and neocolonization, raising the possibility of both community reassembly and healthcare-associated acquisition in selected contexts [13,14]. Despite these insights, evidence remains heterogeneous, and data focused specifically on 0-3-year-old children using quantitative fungal burden alongside standardized oral health assessment remain comparatively limited. Accordingly, this study compared *Candida* carriage and oral health indices in 0-3-year-old children with trans-foramen cleft lip and palate versus age- and sex-matched controls.

## Materials and methods

Study design

A comparative observational study was conducted after approval from the Institutional Ethics Committee. Recruitment for the cleft lip and palate cohort was carried out at the Smile Train Center of Drs. Sudha and Nageswara Rao Siddhartha Institute of Dental Sciences, and the control participants were recruited from the Department of Pediatrics at Dr. Pinnamaneni Siddhartha Institute of Medical Sciences. Written informed consent was obtained from parents or legal guardians in the local language before enrolment. ICE No: IEC/DRS.S&NRSIDS/2023/UG/01. Dated July 28, 2023. This study was completed between August 2023 and July 2024.

Participants and eligibility criteria

Children aged 0 to 3 years were enrolled into two groups using consecutive sampling without gender bias. The study group included 40 children with cleft lip and palate, and the control group included 40 age- and sex-matched children without cleft lip or palate.

Inclusion Criteria

Eligible participants were children aged 0 to 3 years. For the study group, inclusion required the presence of unilateral or bilateral trans-foramen cleft lip and palate. For the control group, inclusion required the absence of a cleft lip or palate, with a match to the study group by age and sex.

Exclusion Criteria

Children were excluded (above three years) from either group if they had received antimicrobial agents, antifungal agents, or immunosuppressive medications within the four-week period preceding oral sample collection.

Oral examination and assessment of oral health

All participants underwent an oral examination under adequate illumination using a mouth mirror, explorer, and Williams periodontal probe. For very young children, the examination was performed with the child seated on a parent or guardian lap to ensure safety and cooperation. Oral hygiene and caries status were assessed using the def index (Gruebbel, 1944 [15]) and the caries severity index (Chosack, 1986 [16]), following World Health Organization criteria as applied in the study protocol.

Sample collection and transport

Oral smear samples were collected using a sterile cotton swab moistened with normal saline. The swab was applied with a standardized twisting motion across the dorsum of the tongue, buccal mucosa, palatal mucosa, and floor of the mouth. Each swab was placed into a sterile tube immediately after collection and transported to the medical mycology laboratory for culture and identification.

Culture procedures and microbiological identification

Swab tips were inoculated into brain heart infusion broth and subsequently streaked onto Sabouraud dextrose agar. Culture plates were incubated at 37°C and observed for up to three days. Growth suggestive of *Candida *was assessed based on colony characteristics and confirmed by wet mount microscopy demonstrating blastospores under a 40× objective.

*C. albicans* was identified using the germ tube test. A yeast suspension from an isolated colony was prepared in 0.5 mL of fresh human serum and incubated at 37°C for up to three hours, followed by microscopic evaluation for germ tube formation under a 40× objective. Non-albicans *Candida *species were differentiated using CHROMagar differential medium according to the protocol [17].

Quantification of fungal burden

Fungal burden was quantified by colony counting using a fixed-volume loop of 4 mm diameter holding 0.005 mL. Colony-forming units per milliliter were calculated by multiplying the colony count by 200.

Statistical analysis

Data were entered into Microsoft Excel and analyzed using IBM SPSS Statistics for Windows, Version 30 (Released 2024; IBM Corp., Armonk, New York, United States). Normality was assessed using the Shapiro-Wilk test. Independent t-tests were used to compare the def index and CSI between groups. Chi-square tests were used for categorical associations, and Fisher exact test was applied when expected cell counts were small. Differences in *Candida *growth category between groups were evaluated using the Mann-Whitney U test. A p-value below 0.05 was considered statistically significant.

## Results

Study participants and baseline distribution

Eighty children aged 0-3 years were enrolled, including 40 children with cleft conditions and 40 age- and sex-matched controls. No child in either group exhibited clinical signs of oral candidiasis. Asymptomatic oral carriage of *Candida* (any growth) was markedly higher in children with cleft conditions (35 (87.5%)) than in controls (8 (20.0%)). In the cleft group,* C. albicans* was the predominant isolate (28 (70.0%)), while non-albicans *Candida *constituted 7 (17.5%). In the control group, *C. albicans* and non-albicans *Candida* were detected in six (15.0%) and two (5.0%) children, respectively. The overall distribution of growth categories (no growth vs. *C. albicans* vs. non-albicans *Candida*) differed significantly between groups (χ2(2) =36.72, p<0.001) (Table 1).

**Table 1:** Distribution of participants in cleft and control groups (n=40 each)

Variable	Category	Cleft group n (%)	Control group n (%)
Age group	0-6 months	20 (50.0)	19 (47.5)
	7 months-1.5 years	16 (40.0)	17 (42.5)
	1.5-3 years	4 (10.0)	4 (10.0)
Gender	Male	23 (57.5)	23 (57.5)
	Female	17 (42.5)	17 (42.5)
*Candida* growth category	No growth	5 (12.5)	32 (80.0)
	Candida albicans	28 (70.0)	6 (15.0)
	Non-albicans *Candida*	7 (17.5)	2 (5.0)

Association of *Candida* growth with age and gender

Within the cleft group, neither age band nor sex showed a statistically significant association with *Candida *growth category. Similarly, within the control group, *Candida* growth category was not significantly associated with age band or sex (Table 2).

**Table 2:** Association of Candida growth category with age group and gender The Pearson chi-square test was used for within-study group associations. A p-value <0.05 was considered statistically significant (p-values <0.001 are also reported where applicable).

Cohort	Factor	Category	No growth n (%)	*Candida albicans* n (%)	*Non-albicans Candida* n (%)	Test statistic	p-value
Cleft (n=40)	Age group	0-6 months (n=20)	4 (20.0)	12 (60.0)	4 (20.0)	χ2(4)=3.54	0.471
		7 months-1.5 years (n=16)	1 (6.2)	12 (75.0)	3 (18.8)		
		1.5-3 years (n=4)	0 (0.0)	4 (100.0)	0 (0.0)		
	Gender	Male (n=23)	5 (21.7)	14 (60.9)	4 (17.4)	χ2(2)=4.34	0.114
		Female (n=17)	0 (0.0)	14 (82.4)	3 (17.6)		
Control (n=40)	Age group	0-6 months (n=19)	16 (84.2)	3 (15.8)	0 (0.0)	χ2(4)=3.82	0.430
		7 months-1.5 years (n=17)	12 (70.6)	3 (17.6)	2 (11.8)		
		1.5-3 years (n=4)	4 (100.0)	0 (0.0)	0 (0.0)		
	Gender	Male (n=23)	17 (73.9)	5 (21.7)	1 (4.3)	χ2(2)=1.94	0.380
		Female (n=17)	15 (88.2)	1 (5.9)	1 (5.9)		

Oral health indices and between-group comparisons

Children with cleft conditions had significantly higher def scores than controls (5.62±0.35 vs 4.83±0.45), with a mean difference of 0.79 (95% CI 0.60-0.96), p<0.001 (Table 3).

**Table 3:** Comparison of def scores between cleft and control groups Independent samples t-test. A p-value <0.05 was considered statistically significant (p-values <0.001 are also reported where applicable). Significant p-values are denoted by *. Mean difference (95% CI): 0.79 (0.60-0.96).

Group	n	Mean ± SD	Std. error mean	Test statistic	p-value
Cleft	40	5.62 ± 0.35	0.055	t(78)=8.76	<0.001*
Control	40	4.83 ± 0.45	0.072		

Children with cleft conditions had significantly higher def scores than controls (5.62±0.35 vs 4.83±0.45), with a mean difference of 0.79 (95% CI 0.60-0.96) (t (78) =8.76, p<0.001) (Table 3). CSI scores were also higher in the cleft group (2.79 ±0.33 vs 1.92±0.28), with a mean difference of 0.87 (95% CI 0.73-1.01) (t (78) =12.71, p<0.001) (Table 4).

**Table 4:** Comparison of CSI scores between cleft and control groups Independent samples t-test. A p-value <0.05 was considered statistically significant (p-values <0.001 are also reported where applicable). Significant p-values are denoted by *. Mean difference (95% CI): 0.87 (0.73-1.01).

Group	n	Mean ± SD	Std. error mean	Test statistic	p-value
Cleft	40	2.79 ± 0.33	0.05	t (78) =12.71	<0.001*
Control	40	1.92 ± 0.28	0.04		

Comparison of overall *Candida* growth outcome between groups

On Mann-Whitney U testing of the ordinal *Candida* growth outcome, the cleft group demonstrated higher ranks (mean rank 53.83) than controls (mean rank 27.18), indicating significantly greater overall culture positivity and species-level growth category in the cleft cohort (U=267, p<0.001) (Table 5).

**Table 5:** Comparison of Candida growth outcome between cleft and control groups Mann-Whitney U test. A p-value <0.05 was considered statistically significant (p-values <0.001 are also reported where applicable). Significant p-values are denoted by *.

Group	n	Mean rank	Test statistic	p-value
Cleft	40	53.83	U=267	<0.001*
Control	40	27.18		

## Discussion

Interpretation of key findings

Asymptomatic oral *Candida* carriage was substantially higher in 0-3-year-old children with cleft lip and palate than in age- and sex-matched controls (35 (87.5%) vs 8 (20.0%)), with a significantly different distribution of growth categories (p<0.001). *C. albicans* predominated in the cleft cohort (28 (70.0%)), while non-albicans *Candida* constituted a smaller but relevant fraction (7 (17.5%)), indicating early diversification. No participant had clinical candidiasis, supporting colonization rather than overt infection as the dominant phenotype. Oral health burden was also higher in the cleft cohort, reflected by greater def scores (mean difference 0.79; 95% CI 0.60-0.96; p<0.001) and higher caries severity index scores (mean difference 0.87; 95% CI 0.73-1.01; p<0.001). Within each cohort, *Candida* growth category was not significantly associated with age band or sex, suggesting that cleft-related ecological factors may outweigh demographic effects during this early-life interval.

Comparison with existing literature

The control carriage rate aligns with background pediatric carriage estimates reported in healthy cohorts, where overall yeast carriage approximates one-quarter and *C. albicans* dominates, with dietary transitions and feeding patterns influencing early-life carriage signals [6]. The elevated cleft-group carriage is concordant with cleft-focused studies showing higher colonization than controls and frequent predominance of *C. albicans*, with some studies reporting increased carriage with bilateral phenotypes and greater surgery exposure [9]. Quantitative work also demonstrates markedly higher CFU burdens in cleft cohorts compared with controls [10]. Infant studies further report very high isolation rates in cleft palate, particularly with plate use, consistent with stagnation niches and hygiene challenges [11]. The observed co-occurrence of high fungal carriage and worse caries-related indices is biologically coherent with evidence that cleft anatomy and care pathways increase plaque accumulation and caries risk [1] and with meta-analytic signals of higher caries odds in cleft populations [2]. Mycobiome-focused synthesis supports early fungal establishment and potential cross-kingdom contributions to cariogenic biofilms [5], while longitudinal infancy data indicate that early *Candida* colonization can precede faster Streptococcus mutans emergence [8].

Explanations for divergence from some studies

The cleft-group prevalence here is at the upper end of published ranges, plausibly due to multi-site sampling that increases detection, the narrow early-life window with intensive cleft-related healthcare contact, and the transforamen CLP phenotype creating pronounced plaque-retentive niches. The lack of age and sex associations is also expected given the restricted 0-3-year range, limited within-band sample sizes, and prior infant cleft cohorts reporting minimal demographic effects during device-based therapy periods [12]. The stronger oral health differences compared with studies reporting similar oral indices despite higher fungal loads may reflect unmeasured heterogeneity in prevention practices, fluoride exposure, diet, and structured dental follow-up across care settings [10]. Temporal dynamics described in perioperative cohorts, including antisepsis-related suppression and subsequent recolonization or acquisition, cannot be evaluated in a cross-sectional design [13,14].

Clinical implications

The findings support integrating early, cleft-adapted oral hygiene counseling and close preventive dental surveillance into routine cleft care, with emphasis on plaque control at cleft-adjacent stagnation sites and any appliance-related niches. Family-centered prevention is justified because maternal plaque burden strongly predicts vertical transmission of *C. albicans*, supporting maternal oral health reinforcement during early cleft care pathways [7]. Asymptomatic carriage alone does not justify routine antifungal treatment; prevention and monitoring are preferable, while antifungal prophylaxis evidence largely applies to high-risk NICU populations rather than outpatient cleft cohorts [18]. Antibiotic stewardship remains relevant given evidence that antibiotics and devices can facilitate *Candida* detection in certain cleft-associated otologic contexts [19].

Strengths and limitations

Strengths include age- and sex-matched controls, prespecified exclusions for recent antimicrobial or immunosuppressive exposure, standardized multi-site oral sampling, species-level differentiation of *C. albicans *versus non-albicans isolates, CFU-based quantification, and concurrent clinical oral health indices that support clinical relevance. Key limitations include the cross-sectional design, a modest sample size that restricts phenotype or exposure subgroup analyses, and reliance on culture-based identification without molecular confirmation or strain-level typing. Recruitment was single-center and clinic-based, which may introduce selection bias and limit generalizability to broader community settings. Several potential confounders were not captured, including feeding patterns, caregiver hygiene practices, socioeconomic factors, daycare exposure, and bacterial co-colonization. Critically, maternal and household carriage data were not collected, and concordance testing between infant and caregiver isolates was not performed, so acquisition pathways, including vertical transmission, cannot be established. Accordingly, findings should be interpreted as associations rather than causal effects [6,7].

## Conclusions

This study adds quantitative, early-childhood evidence that cleft lip and palate is associated with substantially higher asymptomatic oral *Candida* carriage and poorer early caries-related indices, with *C. albicans *predominating and non-albicans species contributing a meaningful minority. The results are largely concordant with prior cleft and pediatric mycobiome literature, while the high prevalence observed here is plausibly explained by intensive multi-site sampling, phenotype-specific anatomic niches, and early-life exposures intrinsic to cleft care. Clinically, the data strengthen the rationale for early, tailored oral hygiene interventions, caregiver education, and integrated dental follow-up within cleft pathways, while supporting conservative management of asymptomatic carriage and emphasizing surveillance and biofilm-oriented prevention over empiric antifungal use.
